# (22*E*,24*R*)-5α-Ergosta-2,22-dien-6-one

**DOI:** 10.1107/S1600536811002388

**Published:** 2011-01-29

**Authors:** Liu-qing Sheng, Fang Zeng, Fei Chen, Chun-nian Xia

**Affiliations:** a Department of Pharmaceutical Science, Jinhua Polytechnic, Jinhua 321007, People’s Republic of China; b College of Pharmaceutical Science, Zhejiang University of Technology, Hangzhou 310032, People’s Republic of China

## Abstract

In the title mol­ecule, C_28_H_44_O, two six-membered rings have regular chair conformations, while the six-membered ring containing the C=C double bond exhibits a distorted chair conformation. The five-membered ring adopts an envelope conformation. In the crystal, weak inter­molecular C—H⋯O inter­actions link mol­ecules into chains along the *b* axis. The absolute configuration was assigned to correspond with that of the known chiral centres in a precursor mol­ecule.

## Related literature

For details of the synthesis, see: McMorris & Patil (1993 ▶). For the crystal structure of the related compound (22*E*,24*R*)-3α,5-cyclo-5α-ergosta-22-en-6-one, see: Sheng *et al.* (2011 ▶).
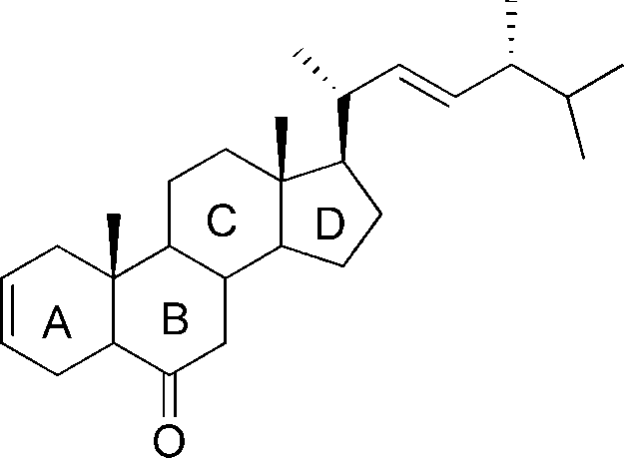


## Experimental

### 

#### Crystal data

C_28_H_44_O
*M* *_r_* = 396.63
Monoclinic, 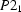

*a* = 9.812 (3) Å
*b* = 7.578 (2) Å
*c* = 16.130 (5) Å
β = 92.832 (4)°
*V* = 1197.8 (6) Å^3^
*Z* = 2
Mo *K*α radiation
μ = 0.06 mm^−1^
*T* = 123 K
0.58 × 0.34 × 0.33 mm

#### Data collection

Rigaku AFC10/Saturn724+ diffractometer
10906 measured reflections
2908 independent reflections
2598 reflections with *I* > 2σ(*I*)
*R*_int_ = 0.030

#### Refinement

*R*[*F* ^2^ > 2σ(*F* ^2^)] = 0.042
*wR*(*F* ^2^) = 0.091
*S* = 1.03
2908 reflections
268 parameters
1 restraint
H-atom parameters constrained
Δρ_max_ = 0.29 e Å^−3^
Δρ_min_ = −0.14 e Å^−3^

### 

Data collection: *CrystalClear* (Rigaku/MSC, 2008 ▶); cell refinement: *CrystalClear*; data reduction: *CrystalClear*; program(s) used to solve structure: *SHELXS97* (Sheldrick, 2008 ▶); program(s) used to refine structure: *SHELXL97* (Sheldrick, 2008 ▶); molecular graphics: *PLATON* (Spek, 2009 ▶); software used to prepare material for publication: *publCIF* (Westrip, 2010 ▶).

## Supplementary Material

Crystal structure: contains datablocks I, global. DOI: 10.1107/S1600536811002388/cv5027sup1.cif

Structure factors: contains datablocks I. DOI: 10.1107/S1600536811002388/cv5027Isup2.hkl

Additional supplementary materials:  crystallographic information; 3D view; checkCIF report

## Figures and Tables

**Table 1:** Hydrogen-bond geometry (Å, °)

*D*—H⋯*A*	*D*—H	H⋯*A*	*D*⋯*A*	*D*—H⋯*A*
C1—H1*A*⋯O1^i^	0.99	2.59	3.574 (3)	173

Symmetry code: (i) 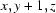.

**Fig. 1.**
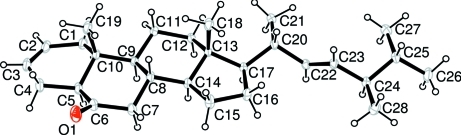
The molecular structure of (I) shown with 30% probability displacement ellipsoids.

## supplementary crystallographic information

### Comment

In continuation of our structural study of brassinolide analogs (Sheng *et al.*, 2011), we present here the crystal structure of the title compound, (I). In (I) (Fig. 1), ring A shows a distorted chair confirmation, and atoms C1, C2, C3 and C4 are coplanar with the r.m.s. deviation of 0.007 (1) Å for the C=C bond; atoms C5 and C10 deviate at 0.329 (4) Å and -0.423 (4) Å from this mean plane, respectively. Rings B and C have regular chair conformation each; while ring D has an envelope conformation. Weak intermolecular C—H···O interactions (Table 1) link the molecules related by translation along the axis *b* into chains.

### Experimental

(22*E*,24*R*)-5α-Ergosta-2,22-dien-6-one was synthesized as a powder according to the known method (McMorris *et al.*, 1993). Crystals of (I) suitable for structure analysis were obtained by slow evaporation from a mixture of acetone and 95% ethanol (volume proportion, 1:1) as colourless prisms.

### Refinement

C-bound H atoms were placed at calculated positions (C—H 0.95–1.00 Å) and constrained to ride on their parent atoms, with*U*_iso_(H) = 1.2-1.5 *U*_eq_(C). Because of negligible anomalous scattering effects, 2305 Friedel pairs were averaged in the refinement. The absolute configuration was assigned to correspond with that of the known chiral centres in a precursor molecule, which remained unchanged during the synthesis of the title compound.

### Crystal data

**Table:**

C_28_H_44_O	*F*(000) = 440
*M**_r_* = 396.63	*D*_x_ = 1.100 Mg m^−^^3^
Monoclinic, *P*2_1_	Mo *K*α radiation, λ = 0.71073 Å
Hall symbol: P 2yb	Cell parameters from 3421 reflections
*a* = 9.812 (3) Å	θ = 3.2–27.5°
*b* = 7.578 (2) Å	µ = 0.06 mm^−^^1^
*c* = 16.130 (5) Å	*T* = 123 K
β = 92.832 (4)°	Block, colourless
*V* = 1197.8 (6) Å^3^	0.58 × 0.34 × 0.33 mm
*Z* = 2	

### Data collection

**Table:**

Rigaku AFC10/Saturn724+ diffractometer	2598 reflections with *I* > 2σ(*I*)
Radiation source: Rotating Anode	*R*_int_ = 0.030
graphite	θ_max_ = 27.5°, θ_min_ = 3.2°
Detector resolution: 28.5714 pixels mm^-1^	*h* = −12→12
phi and ω scans	*k* = −9→9
10906 measured reflections	*l* = −20→20
2908 independent reflections	

### Refinement

**Table:**

Refinement on *F*^2^	Primary atom site location: structure-invariant direct methods
Least-squares matrix: full	Secondary atom site location: difference Fourier map
*R*[*F*^2^ > 2σ(*F*^2^)] = 0.042	Hydrogen site location: inferred from neighbouring sites
*wR*(*F*^2^) = 0.091	H-atom parameters constrained
*S* = 1.03	*w* = 1/[σ^2^(*F*_o_^2^) + (0.0346*P*)^2^ + 0.296*P*] where *P* = (*F*_o_^2^ + 2*F*_c_^2^)/3
2908 reflections	(Δ/σ)_max_ < 0.001
268 parameters	Δρ_max_ = 0.29 e Å^−^^3^
1 restraint	Δρ_min_ = −0.14 e Å^−^^3^

### Special details

**Table:**

Geometry. All esds (except the esd in the dihedral angle between two l.s. planes) are estimated using the full covariance matrix. The cell esds are taken into account individually in the estimation of esds in distances, angles and torsion angles; correlations between esds in cell parameters are only used when they are defined by crystal symmetry. An approximate (isotropic) treatment of cell esds is used for estimating esds involving l.s. planes.
Refinement. Refinement of F^2^ against ALL reflections. The weighted R-factor wR and goodness of fit S are based on F^2^, conventional R-factors R are based on F, with F set to zero for negative F^2^. The threshold expression of F^2^ > 2sigma(F^2^) is used only for calculating R-factors(gt) etc. and is not relevant to the choice of reflections for refinement. R-factors based on F^2^ are statistically about twice as large as those based on F, and R- factors based on ALL data will be even larger.

### Fractional atomic coordinates and isotropic or equivalent isotropic displacement parameters (Å^2^)

**Table:**

	*x*	*y*	*z*	*U*_iso_*/*U*_eq_	
O1	0.41104 (19)	−0.0462 (3)	0.15251 (10)	0.0498 (5)	
C1	0.5886 (2)	0.5431 (3)	0.17436 (13)	0.0335 (5)	
H1A	0.5444	0.6606	0.1729	0.040*	
H1B	0.6598	0.5438	0.2201	0.040*	
C2	0.6558 (2)	0.5130 (4)	0.09327 (13)	0.0374 (6)	
H2	0.7045	0.6082	0.0704	0.045*	
C3	0.6508 (2)	0.3625 (4)	0.05232 (13)	0.0378 (6)	
H3	0.6940	0.3562	0.0010	0.045*	
C4	0.5807 (2)	0.2008 (4)	0.08234 (13)	0.0377 (6)	
H4A	0.6414	0.0975	0.0768	0.045*	
H4B	0.4967	0.1797	0.0472	0.045*	
C5	0.5433 (2)	0.2187 (3)	0.17289 (12)	0.0295 (5)	
H5	0.6303	0.2082	0.2075	0.035*	
C6	0.4516 (2)	0.0705 (3)	0.19923 (13)	0.0332 (5)	
C7	0.4118 (2)	0.0762 (3)	0.28843 (13)	0.0324 (5)	
H7A	0.4925	0.0480	0.3253	0.039*	
H7B	0.3416	−0.0150	0.2970	0.039*	
C8	0.3559 (2)	0.2571 (3)	0.31218 (12)	0.0258 (4)	
H8	0.2648	0.2742	0.2826	0.031*	
C9	0.4513 (2)	0.4064 (3)	0.28672 (11)	0.0243 (4)	
H9	0.5407	0.3842	0.3174	0.029*	
C10	0.4813 (2)	0.4025 (3)	0.19277 (12)	0.0269 (5)	
C11	0.4016 (2)	0.5869 (3)	0.31708 (12)	0.0300 (5)	
H11A	0.3167	0.6192	0.2850	0.036*	
H11B	0.4712	0.6773	0.3060	0.036*	
C12	0.3740 (2)	0.5889 (3)	0.41042 (12)	0.0280 (5)	
H12A	0.4615	0.5743	0.4430	0.034*	
H12B	0.3352	0.7047	0.4250	0.034*	
C13	0.27536 (19)	0.4423 (3)	0.43412 (11)	0.0232 (4)	
C14	0.3378 (2)	0.2678 (3)	0.40546 (12)	0.0233 (4)	
H14	0.4318	0.2625	0.4323	0.028*	
C15	0.2560 (2)	0.1243 (3)	0.44678 (14)	0.0361 (5)	
H15A	0.3107	0.0152	0.4548	0.043*	
H15B	0.1716	0.0962	0.4132	0.043*	
C16	0.2221 (2)	0.2060 (3)	0.53128 (13)	0.0314 (5)	
H16A	0.2730	0.1444	0.5772	0.038*	
H16B	0.1232	0.1960	0.5400	0.038*	
C17	0.26526 (19)	0.4034 (3)	0.52833 (11)	0.0220 (4)	
H17	0.3596	0.4119	0.5544	0.026*	
C18	0.1323 (2)	0.4742 (4)	0.39396 (12)	0.0391 (6)	
H18A	0.1363	0.4695	0.3334	0.059*	
H18B	0.0994	0.5906	0.4104	0.059*	
H18C	0.0698	0.3830	0.4124	0.059*	
C19	0.3521 (2)	0.4340 (4)	0.13685 (12)	0.0396 (6)	
H19A	0.3116	0.5477	0.1508	0.059*	
H19B	0.2862	0.3393	0.1455	0.059*	
H19C	0.3761	0.4349	0.0786	0.059*	
C20	0.1723 (2)	0.5195 (3)	0.57980 (12)	0.0261 (4)	
H20	0.0767	0.5067	0.5562	0.031*	
C21	0.2101 (2)	0.7153 (3)	0.57749 (13)	0.0337 (5)	
H21A	0.1588	0.7797	0.6184	0.051*	
H21B	0.1876	0.7627	0.5220	0.051*	
H21C	0.3082	0.7289	0.5906	0.051*	
C22	0.17560 (19)	0.4580 (3)	0.66901 (11)	0.0250 (4)	
H22	0.2625	0.4501	0.6974	0.030*	
C23	0.06861 (19)	0.4144 (3)	0.71088 (11)	0.0257 (4)	
H23	−0.0176	0.4147	0.6813	0.031*	
C24	0.0705 (2)	0.3640 (3)	0.80131 (12)	0.0247 (4)	
H24	0.1667	0.3725	0.8245	0.030*	
C25	−0.0184 (2)	0.4927 (3)	0.85049 (13)	0.0304 (5)	
H25	−0.1156	0.4767	0.8303	0.037*	
C26	−0.0087 (3)	0.4528 (4)	0.94378 (13)	0.0490 (7)	
H26A	0.0863	0.4646	0.9648	0.073*	
H26B	−0.0402	0.3321	0.9533	0.073*	
H26C	−0.0660	0.5361	0.9729	0.073*	
C27	0.0205 (3)	0.6848 (3)	0.83604 (15)	0.0375 (6)	
H27A	−0.0353	0.7619	0.8694	0.056*	
H27B	0.0048	0.7138	0.7771	0.056*	
H27C	0.1172	0.7021	0.8523	0.056*	
C28	0.0230 (3)	0.1728 (3)	0.81002 (14)	0.0368 (6)	
H28A	−0.0737	0.1638	0.7922	0.055*	
H28B	0.0354	0.1360	0.8681	0.055*	
H28C	0.0769	0.0961	0.7753	0.055*	

### Atomic displacement parameters (Å^2^)

**Table:**

	*U*^11^	*U*^22^	*U*^33^	*U*^12^	*U*^13^	*U*^23^
O1	0.0724 (12)	0.0452 (11)	0.0322 (9)	−0.0115 (10)	0.0075 (8)	−0.0167 (8)
C1	0.0334 (11)	0.0418 (14)	0.0261 (11)	0.0016 (10)	0.0079 (9)	0.0050 (9)
C2	0.0312 (11)	0.0556 (16)	0.0260 (10)	0.0020 (11)	0.0083 (9)	0.0100 (11)
C3	0.0304 (11)	0.0628 (18)	0.0207 (10)	0.0059 (11)	0.0053 (9)	0.0023 (10)
C4	0.0363 (12)	0.0549 (16)	0.0222 (10)	0.0028 (11)	0.0063 (9)	−0.0067 (10)
C5	0.0288 (11)	0.0409 (13)	0.0191 (9)	0.0024 (10)	0.0025 (8)	−0.0039 (9)
C6	0.0398 (12)	0.0349 (13)	0.0251 (11)	0.0022 (10)	0.0033 (9)	−0.0066 (9)
C7	0.0399 (12)	0.0311 (12)	0.0268 (10)	−0.0042 (10)	0.0072 (9)	−0.0064 (9)
C8	0.0255 (10)	0.0324 (12)	0.0197 (9)	−0.0005 (9)	0.0023 (8)	−0.0034 (8)
C9	0.0236 (9)	0.0301 (11)	0.0194 (9)	0.0024 (9)	0.0020 (7)	0.0016 (8)
C10	0.0247 (10)	0.0380 (13)	0.0183 (9)	0.0041 (9)	0.0026 (8)	0.0001 (8)
C11	0.0364 (11)	0.0296 (12)	0.0249 (10)	0.0058 (10)	0.0104 (9)	0.0054 (9)
C12	0.0358 (11)	0.0239 (11)	0.0254 (10)	0.0026 (9)	0.0111 (9)	0.0004 (8)
C13	0.0221 (9)	0.0296 (11)	0.0181 (8)	0.0023 (8)	0.0031 (7)	−0.0011 (8)
C14	0.0246 (10)	0.0257 (11)	0.0200 (9)	−0.0045 (8)	0.0037 (7)	−0.0020 (8)
C15	0.0452 (13)	0.0335 (13)	0.0307 (11)	−0.0161 (11)	0.0128 (10)	−0.0080 (10)
C16	0.0367 (12)	0.0320 (13)	0.0262 (10)	−0.0097 (10)	0.0101 (9)	−0.0033 (9)
C17	0.0211 (9)	0.0264 (11)	0.0185 (9)	−0.0014 (8)	0.0024 (7)	−0.0010 (8)
C18	0.0277 (11)	0.0665 (18)	0.0231 (10)	0.0095 (11)	0.0013 (8)	0.0017 (11)
C19	0.0307 (11)	0.0685 (18)	0.0196 (9)	0.0104 (12)	0.0023 (8)	0.0052 (11)
C20	0.0207 (9)	0.0364 (12)	0.0214 (9)	0.0020 (9)	0.0030 (7)	−0.0001 (9)
C21	0.0399 (12)	0.0331 (12)	0.0292 (11)	0.0106 (10)	0.0110 (9)	0.0005 (9)
C22	0.0227 (9)	0.0316 (12)	0.0206 (9)	0.0008 (9)	0.0012 (7)	−0.0043 (8)
C23	0.0254 (10)	0.0306 (11)	0.0211 (9)	0.0010 (9)	0.0009 (8)	−0.0015 (8)
C24	0.0233 (10)	0.0306 (12)	0.0204 (10)	−0.0026 (8)	0.0026 (8)	−0.0008 (8)
C25	0.0266 (10)	0.0385 (13)	0.0266 (10)	−0.0015 (9)	0.0064 (8)	−0.0039 (9)
C26	0.0652 (16)	0.0556 (17)	0.0278 (11)	−0.0009 (15)	0.0192 (11)	−0.0005 (12)
C27	0.0403 (13)	0.0354 (13)	0.0374 (12)	0.0047 (10)	0.0066 (10)	−0.0054 (10)
C28	0.0439 (14)	0.0339 (13)	0.0326 (12)	−0.0090 (10)	0.0021 (10)	0.0014 (10)

### Geometric parameters (Å, °)

**Table:**

O1—C6	1.216 (3)	C15—H15A	0.9900
C1—C2	1.511 (3)	C15—H15B	0.9900
C1—C10	1.537 (3)	C16—C17	1.556 (3)
C1—H1A	0.9900	C16—H16A	0.9900
C1—H1B	0.9900	C16—H16B	0.9900
C2—C3	1.318 (4)	C17—C20	1.539 (3)
C2—H2	0.9500	C17—H17	1.0000
C3—C4	1.497 (4)	C18—H18A	0.9800
C3—H3	0.9500	C18—H18B	0.9800
C4—C5	1.529 (3)	C18—H18C	0.9800
C4—H4A	0.9900	C19—H19A	0.9800
C4—H4B	0.9900	C19—H19B	0.9800
C5—C6	1.513 (3)	C19—H19C	0.9800
C5—C10	1.559 (3)	C20—C22	1.511 (3)
C5—H5	1.0000	C20—C21	1.530 (3)
C6—C7	1.510 (3)	C20—H20	1.0000
C7—C8	1.532 (3)	C21—H21A	0.9800
C7—H7A	0.9900	C21—H21B	0.9800
C7—H7B	0.9900	C21—H21C	0.9800
C8—C14	1.526 (3)	C22—C23	1.318 (3)
C8—C9	1.537 (3)	C22—H22	0.9500
C8—H8	1.0000	C23—C24	1.507 (3)
C9—C11	1.540 (3)	C23—H23	0.9500
C9—C10	1.558 (2)	C24—C28	1.531 (3)
C9—H9	1.0000	C24—C25	1.552 (3)
C10—C19	1.538 (3)	C24—H24	1.0000
C11—C12	1.543 (3)	C25—C27	1.526 (3)
C11—H11A	0.9900	C25—C26	1.533 (3)
C11—H11B	0.9900	C25—H25	1.0000
C12—C13	1.534 (3)	C26—H26A	0.9800
C12—H12A	0.9900	C26—H26B	0.9800
C12—H12B	0.9900	C26—H26C	0.9800
C13—C18	1.536 (3)	C27—H27A	0.9800
C13—C14	1.539 (3)	C27—H27B	0.9800
C13—C17	1.556 (2)	C27—H27C	0.9800
C14—C15	1.525 (3)	C28—H28A	0.9800
C14—H14	1.0000	C28—H28B	0.9800
C15—C16	1.548 (3)	C28—H28C	0.9800
			
C2—C1—C10	113.2 (2)	C16—C15—H15A	111.0
C2—C1—H1A	108.9	C14—C15—H15B	111.0
C10—C1—H1A	108.9	C16—C15—H15B	111.0
C2—C1—H1B	108.9	H15A—C15—H15B	109.0
C10—C1—H1B	108.9	C15—C16—C17	106.68 (16)
H1A—C1—H1B	107.7	C15—C16—H16A	110.4
C3—C2—C1	123.9 (2)	C17—C16—H16A	110.4
C3—C2—H2	118.0	C15—C16—H16B	110.4
C1—C2—H2	118.0	C17—C16—H16B	110.4
C2—C3—C4	123.5 (2)	H16A—C16—H16B	108.6
C2—C3—H3	118.2	C20—C17—C13	119.12 (16)
C4—C3—H3	118.2	C20—C17—C16	111.31 (16)
C3—C4—C5	111.8 (2)	C13—C17—C16	104.04 (16)
C3—C4—H4A	109.3	C20—C17—H17	107.3
C5—C4—H4A	109.3	C13—C17—H17	107.3
C3—C4—H4B	109.3	C16—C17—H17	107.3
C5—C4—H4B	109.3	C13—C18—H18A	109.5
H4A—C4—H4B	107.9	C13—C18—H18B	109.5
C6—C5—C4	112.09 (19)	H18A—C18—H18B	109.5
C6—C5—C10	111.21 (16)	C13—C18—H18C	109.5
C4—C5—C10	112.96 (19)	H18A—C18—H18C	109.5
C6—C5—H5	106.7	H18B—C18—H18C	109.5
C4—C5—H5	106.7	C10—C19—H19A	109.5
C10—C5—H5	106.7	C10—C19—H19B	109.5
O1—C6—C7	121.3 (2)	H19A—C19—H19B	109.5
O1—C6—C5	123.2 (2)	C10—C19—H19C	109.5
C7—C6—C5	115.59 (18)	H19A—C19—H19C	109.5
C6—C7—C8	112.10 (19)	H19B—C19—H19C	109.5
C6—C7—H7A	109.2	C22—C20—C21	109.17 (18)
C8—C7—H7A	109.2	C22—C20—C17	110.62 (17)
C6—C7—H7B	109.2	C21—C20—C17	112.97 (17)
C8—C7—H7B	109.2	C22—C20—H20	108.0
H7A—C7—H7B	107.9	C21—C20—H20	108.0
C14—C8—C7	110.76 (18)	C17—C20—H20	108.0
C14—C8—C9	109.04 (16)	C20—C21—H21A	109.5
C7—C8—C9	111.18 (16)	C20—C21—H21B	109.5
C14—C8—H8	108.6	H21A—C21—H21B	109.5
C7—C8—H8	108.6	C20—C21—H21C	109.5
C9—C8—H8	108.6	H21A—C21—H21C	109.5
C8—C9—C11	111.22 (15)	H21B—C21—H21C	109.5
C8—C9—C10	113.13 (17)	C23—C22—C20	125.81 (18)
C11—C9—C10	113.82 (17)	C23—C22—H22	117.1
C8—C9—H9	106.0	C20—C22—H22	117.1
C11—C9—H9	106.0	C22—C23—C24	125.98 (18)
C10—C9—H9	106.0	C22—C23—H23	117.0
C1—C10—C19	109.40 (19)	C24—C23—H23	117.0
C1—C10—C9	109.81 (17)	C23—C24—C28	109.84 (17)
C19—C10—C9	112.18 (15)	C23—C24—C25	110.81 (17)
C1—C10—C5	107.53 (16)	C28—C24—C25	111.51 (17)
C19—C10—C5	109.67 (19)	C23—C24—H24	108.2
C9—C10—C5	108.13 (16)	C28—C24—H24	108.2
C9—C11—C12	113.11 (18)	C25—C24—H24	108.2
C9—C11—H11A	109.0	C27—C25—C26	109.5 (2)
C12—C11—H11A	109.0	C27—C25—C24	111.72 (17)
C9—C11—H11B	109.0	C26—C25—C24	111.6 (2)
C12—C11—H11B	109.0	C27—C25—H25	107.9
H11A—C11—H11B	107.8	C26—C25—H25	107.9
C13—C12—C11	112.29 (18)	C24—C25—H25	107.9
C13—C12—H12A	109.1	C25—C26—H26A	109.5
C11—C12—H12A	109.1	C25—C26—H26B	109.5
C13—C12—H12B	109.1	H26A—C26—H26B	109.5
C11—C12—H12B	109.1	C25—C26—H26C	109.5
H12A—C12—H12B	107.9	H26A—C26—H26C	109.5
C12—C13—C18	110.85 (19)	H26B—C26—H26C	109.5
C12—C13—C14	106.36 (15)	C25—C27—H27A	109.5
C18—C13—C14	112.21 (18)	C25—C27—H27B	109.5
C12—C13—C17	116.88 (16)	H27A—C27—H27B	109.5
C18—C13—C17	109.92 (15)	C25—C27—H27C	109.5
C14—C13—C17	100.14 (16)	H27A—C27—H27C	109.5
C15—C14—C8	118.85 (17)	H27B—C27—H27C	109.5
C15—C14—C13	104.80 (16)	C24—C28—H28A	109.5
C8—C14—C13	114.15 (17)	C24—C28—H28B	109.5
C15—C14—H14	106.0	H28A—C28—H28B	109.5
C8—C14—H14	106.0	C24—C28—H28C	109.5
C13—C14—H14	106.0	H28A—C28—H28C	109.5
C14—C15—C16	103.90 (17)	H28B—C28—H28C	109.5
C14—C15—H15A	111.0		
			
C10—C1—C2—C3	−16.3 (3)	C11—C12—C13—C14	55.7 (2)
C1—C2—C3—C4	−1.6 (4)	C11—C12—C13—C17	166.48 (17)
C2—C3—C4—C5	−12.2 (3)	C7—C8—C14—C15	−52.7 (3)
C3—C4—C5—C6	170.17 (19)	C9—C8—C14—C15	−175.30 (18)
C3—C4—C5—C10	43.6 (2)	C7—C8—C14—C13	−177.17 (16)
C4—C5—C6—O1	−1.4 (3)	C9—C8—C14—C13	60.2 (2)
C10—C5—C6—O1	126.1 (2)	C12—C13—C14—C15	167.55 (17)
C4—C5—C6—C7	178.9 (2)	C18—C13—C14—C15	−71.1 (2)
C10—C5—C6—C7	−53.6 (2)	C17—C13—C14—C15	45.47 (18)
O1—C6—C7—C8	−129.2 (2)	C12—C13—C14—C8	−60.7 (2)
C5—C6—C7—C8	50.5 (3)	C18—C13—C14—C8	60.6 (2)
C6—C7—C8—C14	−171.04 (17)	C17—C13—C14—C8	177.18 (16)
C6—C7—C8—C9	−49.7 (2)	C8—C14—C15—C16	−163.18 (19)
C14—C8—C9—C11	−52.8 (2)	C13—C14—C15—C16	−34.2 (2)
C7—C8—C9—C11	−175.21 (17)	C14—C15—C16—C17	9.2 (2)
C14—C8—C9—C10	177.62 (16)	C12—C13—C17—C20	82.5 (2)
C7—C8—C9—C10	55.2 (2)	C18—C13—C17—C20	−44.9 (3)
C2—C1—C10—C19	−74.4 (2)	C14—C13—C17—C20	−163.16 (17)
C2—C1—C10—C9	162.09 (17)	C12—C13—C17—C16	−152.84 (18)
C2—C1—C10—C5	44.7 (2)	C18—C13—C17—C16	79.7 (2)
C8—C9—C10—C1	−174.10 (17)	C14—C13—C17—C16	−38.54 (18)
C11—C9—C10—C1	57.7 (2)	C15—C16—C17—C20	148.14 (17)
C8—C9—C10—C19	64.0 (2)	C15—C16—C17—C13	18.6 (2)
C11—C9—C10—C19	−64.2 (2)	C13—C17—C20—C22	179.15 (17)
C8—C9—C10—C5	−57.0 (2)	C16—C17—C20—C22	58.1 (2)
C11—C9—C10—C5	174.73 (17)	C13—C17—C20—C21	−58.1 (2)
C6—C5—C10—C1	172.97 (17)	C16—C17—C20—C21	−179.16 (18)
C4—C5—C10—C1	−60.0 (2)	C21—C20—C22—C23	110.1 (2)
C6—C5—C10—C19	−68.2 (2)	C17—C20—C22—C23	−125.0 (2)
C4—C5—C10—C19	58.9 (2)	C20—C22—C23—C24	−175.9 (2)
C6—C5—C10—C9	54.5 (2)	C22—C23—C24—C28	−116.4 (2)
C4—C5—C10—C9	−178.51 (17)	C22—C23—C24—C25	119.9 (2)
C8—C9—C11—C12	51.5 (2)	C23—C24—C25—C27	−52.4 (2)
C10—C9—C11—C12	−179.31 (17)	C28—C24—C25—C27	−175.10 (19)
C9—C11—C12—C13	−54.1 (2)	C23—C24—C25—C26	−175.45 (19)
C11—C12—C13—C18	−66.5 (2)	C28—C24—C25—C26	61.9 (2)

### Hydrogen-bond geometry (Å, °)

**Table:**

*D*—H···*A*	*D*—H	H···*A*	*D*···*A*	*D*—H···*A*
C1—H1A···O1^i^	0.99	2.59	3.574 (3)	173

Symmetry codes: (i) *x*, *y*+1, *z*.
